# The Impact of the Combined Administration of 1MeTIQ and MK-801 on Cell Viability, Oxidative Stress Markers, and Glutamate Release in the Rat Hippocampus

**DOI:** 10.1007/s12640-021-00428-9

**Published:** 2021-10-19

**Authors:** Agnieszka Wąsik, Magdalena Białoń, Danuta Jantas, Marcelina Żarnowska

**Affiliations:** 1 Department of Neurochemistry, Maj Institute of Pharmacology PAS, Krakow, Poland; 2 Department of Experimental Neuroendocrinology, Maj Institute of Pharmacology PAS, Krakow, Poland

**Keywords:** 1-Methyl-1,2,3,4-tetrahydroisoquinoline (1MeTIQ), MK-801, Antioxidant enzymes, Cytotoxicity, Glutamate release

## Abstract

MK-801, as an N-methyl-D-aspartate (NMDA) receptor inhibitor, causes elevation in glutamate release, which may lead to an increase in excitotoxicity, oxidative stress and, consequently, cell death. 1-Methyl-1,2,3,4-tetrahydroisoquinoline (1MeTIQ) shows antioxidant activity. The aim of the present study was to evaluate the effect of combined treatment with 1MeTIQ and MK-801 on cell viability, antioxidant enzyme activity, and glutamate release in the rat hippocampus. Cytotoxicity was measured using lactate dehydrogenase leakage assay (LDH) and the methyl tetrazolium (MTT) assay; antioxidant enzyme activity (glutathione peroxidase (GPx), glutathione reductase (GR), superoxide dismutase (SOD), and catalase (CAT)) were measured by ELISA kits. The release of glutamate in the rat hippocampus was measured using in vivo microdialysis methodology. An in vitro study showed that MK-801 induced cell death in a concentration-dependent manner and that 1MeTIQ partially reduced this adverse effect of MK-801. An ex vivo study indicated that MK-801 produced an increase in antioxidant enzyme activity (GPx, GR, and SOD), whereas coadministration of MK-801 and 1MeTIQ restored the activity of these enzymes to the control level. An in vivo microdialysis study demonstrated that combined treatment with both drugs decreased the release of glutamate in the rat hippocampus. The above results revealed that 1MeTIQ shows limited neuroprotective activity under conditions of glutamate-induced neurotoxicity.

## Introduction

NMDA receptors are associated with memory, learning, cognition and synaptic plasticity (Dore et al. 2017; Fouad et al. 2018). Dysregulation or hypofunction of NMDA receptors, particularly in the hippocampus, may play a key role in the pathogenesis of schizophrenia (Dore et al. 2017). In schizophrenia, disturbed glutamate release is observed in both the frontal cortex and the hippocampus (McCutcheon et al. 2020) Both of these brain structures play a very important role in memory and learning processes (Buckner et al. 1999; Fletcher and Henson 2001; Carr et al. 2011; Guo et al. 2019). NMDA receptor inhibitors such as ketamine and MK-801 are widely used to model schizophrenia in animals. After administration of these compounds, rodents develop cognitive disorders and symptoms resembling positive and negative symptoms of schizophrenia (Mansbach and Geyer 1989; Zhou et al. 2020). MK-801, as an NMDA receptor inhibitor, causes a number of changes in the functioning of neurons, including an increase in glutamate release, which may lead to an increase in excitotoxicity, oxidative stress and, consequently, cell death. Excessive activation of the NMDA receptor causes powerful release of calcium ions, inducing excitotoxicity and, consequently, cell death (Ju and Cui 2016). Another cause of neuronal death is oxidative stress, which is observed in schizophrenia patients (Mahadik and Mukherjee 1996). Reactive oxygen species (ROS) serve as a common initiator of the apoptotic process (Matés 2000). Some authors have indicated that MK-801 induces apoptosis (Zhang et al. 1996; Bueno et al. 2003), and the effect is related to the dosage of MK-801, brain regions, and rodent sex. Biochemical assays such as the lactate dehydrogenase leakage (LDH) and the methyl tetrazolium (MTT) reduction assays are widely used in in vitro toxicology studies to estimate cell toxicity and viability, respectively. LDH and the MTT assay are the most commonly employed for the detection of cytotoxicity or cell viability following exposure to toxic substances (Fotakis and Timbrell 2006). Ozyurt et al. (2007) revealed that MK-801 induces oxidative injury, including tissue lipid peroxidation and antioxidant enzyme activation. Antioxidant enzymes such as glutathione peroxidase (GPx), glutathione reductase (GR), superoxide dismutase (SOD), and catalase (CAT) have complementary activities in the antioxidative defense system. SOD can selectively scavenge O_2_^−^ by catalyzing its dismutation to H_2_O_2_ and oxygen (O_2_), while CAT the conversion of H_2_O_2_ to water and oxygen (Matés and Sánchez-Jiménez 1999). Increased antioxidant enzyme activity may reflect a preceding cellular oxidative stress or serve as a compensatory mechanism.

Our earlier studies demonstrated that 1-methyl-1,2,3,4-tetrahydroisoquinoline (1MeTIQ) shows neuroprotective activity both in vitro (Antkiewicz-Michaluk et al. 2006) and in vivo in several animal models of PD (Antkiewicz-Michaluk et al. 2003, 2004, 2014; Wąsik et al. 2016a, b; Wąsik and Antkiewicz-Michaluk 2017). 1MeTIQ acts as a reversible monoamine oxidase (MAO) inhibitor, blocks free radical formation generated during dopamine oxidation via the Fenton reaction and, consequently, exhibits essential antioxidant properties (Singer and Ramsay 1995; Patsenka and Antkiewicz-Michaluk 2004; Antkiewicz-Michaluk et al. 2006). These results indicated that 1MeTIQ exerts antioxidant properties and acts as a natural scavenger of free radicals in the rodent brain. Moreover, 1MeTIQ antagonized the kainate-induced release of glutamate and aspartate in the rat frontal cortex (Antkiewicz-Michaluk et al. 2006). 1MeTIQ affects both dopaminergic and glutamatergic systems in the brain, and both of these systems play an important role in schizophrenia (Seeman 1987; Kristiansen et al. 2007; Stone et al. 2007). As demonstrated earlier, 1MeTIQ reversed the enhancement of dopaminergic transmission produced by MK-801 in the striatum and glutamate release in the frontal cortex. Behavioral studies have shown that 1MeTIQ completely antagonizes MK-801-induced locomotor hyperactivity; however, it does not antagonize disruption of PPI or working memory impairment evoked by MK-801, which may serve as a model of negative symptoms of schizophrenia (Pietraszek et al. 2009). 1MeTIQ shows antiradical and limited neuroprotective activity under conditions of glutamate-induced neurotoxicity not mediated by NMDA receptors. Importantly, 1MeTIQ also inhibits general excitation caused by the release of excitatory amino acids and is able to inhibit NMDA receptors and to block glutamate-induced calcium entry into neurons (Antkiewicz-Michaluk et al. 2006).

The purpose of the current research was to evaluate the effect of combined treatment with 1MeTIQ and MK-801 on cell viability in mouse primary neuronal cell cultures and the activity of antioxidant enzymes in the rat frontal cortex (Fcx) and hippocampus (HIP). In addition, the effect of the administration of both drugs on the extraneuronal concentration of glutamate in the rat hippocampus was measured using in vivo microdialysis methodology.

## Materials and Methods

### In Vitro Study

#### Drugs and Reagents

**Fig. 1:**
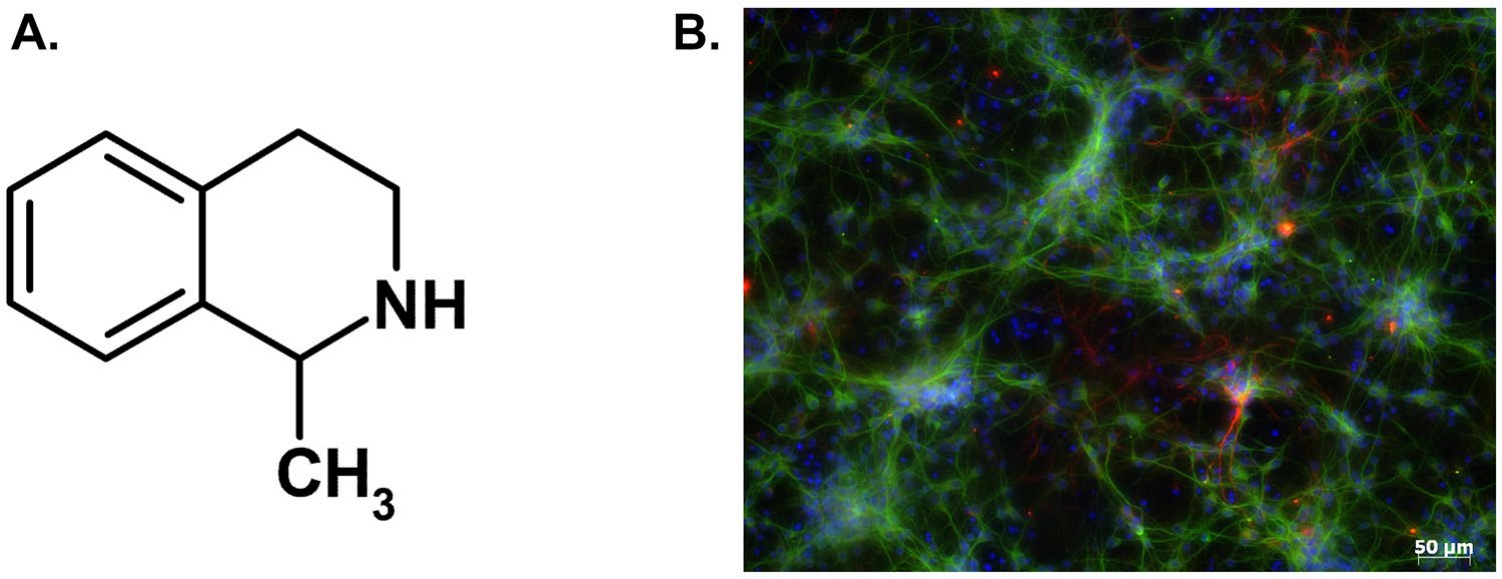
(Panel **A**) Chemical structure of 1-methyl-1,2,3,4-tetrahydroisoquinoline (1MeTIQ). (Panel **B**) Representative image of 3 DIV neuronal cells stained with neuronal (antiMAP2, green) and astrocyte (antiGFAP, red) markers and contrastained with nuclear markers (Hoechst 33,342, blue)

( +)-MK-801 hydrogen maleate was obtained from Sigma-Aldrich (Sigma-Aldrich Chemie GmbH, Germany). 1-Methyl-1,2,3,4-tetrahydroisoquinoline (1MeTIQ; Fig. 1A) was synthetized in the Department of Drug Chemistry (Maj Institute of Pharmacology Polish Academy of Sciences). Neurobasal A medium, fetal bovine serum (FBS), and supplement B27 (without antioxidants) were purchased from Gibco (Invitrogen, Poisley, UK). The Cytotoxicity Detection Kit was from Roche Diagnostic (Mannheim, Germany). Mouse antiMAP-2 (sc-51669) and rabbit antiGFAP (#G9269) were purchased from Santa Cruz Biotech. and Sigma-Aldrich, respectively. Fluorescently labeled secondary antibodies (antimouse Alexa Fluor®488 and antirabbit Alexa Fluor®568) were purchased from Molecular Probes (Invitrogen). All other reagents were from Sigma-Aldrich.

#### Primary Neuronal Cell Cultures

Primary neuronal cell cultures were generated from the forebrains of Swiss mouse embryos (15 days of gestation, Charles River), according to the procedure described previously (Jantas et al. 2018). The isolated cells were suspended and maintained in a Neurobasal medium supplemented with B27 (without antioxidants) and antibiotics with medium exchange every 2 days. The cultures were maintained at 37 °C in a humidified atmosphere containing 5% CO_2_ for 3 days prior to experimentation. The glial content in neuronal cell cultures was verified by dual antiMAP2 (neuronal marker) and antiGFAP (astrocyte marker) immunocytochemistry, where the number of astrocytes) did not exceed 10% (Fig. 1B). The protocol for generating the primary neuronal cell cultures was in accordance with local and international guidelines on the ethical use of animals. Animal care followed official governmental guidelines, and all efforts were made to minimize the number of animals used and their suffering.

#### Cell Treatment

First, the effective concentrations and time of cell exposure to MK-801 (50–200 μM) to induce significant cell damage at 3 DIV (*days *in vitro) were established after 24 and 48 h of treatment. Next, the effect of 1MeTIQ (50, 100, and 500 μM) given alone to cell culture for 24 h was investigated to verify 1MeTIQ biosafety for neuronal cell cultures. For the study of the neuroprotective potential of 1MeTIQ, 3 DIV cell cultures were co-treated with 1MeTIQ (10, 50, 100, 250, and 500 μM) and MK-801 (200 μM) for 24 h.

#### MTT Reduction Assay

Cell viability was quantified using a tetrazolium salt colorimetric assay with 3-[4,5-dimethylthiazol-2-yl]-2,5-diphenyltetrazolium bromide (MTT), as described previously (Jantas et al. 2018). The absorbance of each sample was measured at a wavelength of 570 nm using a microplate reader (Infinite M200 PRO, Tecan). The data were normalized to the vehicle-treated cells (100%) and expressed as a percent of the control ± SEM established from 2 to 3 independent experiments with 2–4 replicates.

#### LDH Release Assay

The level of cytotoxicity after cell treatment was estimated by measurement of lactate dehydrogenase (LDH) released from cells into the culture media using the Cytotoxicity Detection Kit (Roche) as described previously (Jantas et al. 2018). The absorbance of each sample was measured at a wavelength of 490 nm using a microplate reader (Infinite M200 PRO, Tecan). The data from 2 to 3 independent experiments with 2–4 replicates were normalized to the Triton X100-treated cells (100%) and expressed as a percent of the total ± SEM.

#### Immunocytochemistry and Hoechst 33,342 Staining

The purity of primary neuronal cell cultures and the morphological changes after cell treatment in particular cell types were determined by immunocytochemistry, as described previously (Jantas et al. 2018). The samples from 3 DIV neuronal cell cultures after fixation, permeabilization and blocking were incubated overnight with neuronal (antiMAP-2, 1:250) and astrocyte (antiGFAP, 1:500**)**-specific antibodies followed by 60 min labeling with secondary antibodies (antimouse Alexa Fluor®488 and antirabbit Alexa Fluor®568, dilution 1:500). The nuclei were visualized by staining with Hoechst 33,342 dye, as described previously (Jantas et al. 2018). Next, the samples were mounted with a ProLong®Gold antifade reagent (Invitrogen, USA), and probes were examined using a fluorescence AxioObserver microscope (Carl Zeiss, Germany) with excitation wavelengths of 355 nm (Hoechst 33,342), 470 nm (Alexa®488), and 555 nm (Alexa®568). Five microphotographs for each panel were taken for each tested group in duplicate using a black–white camera (Axio-CamMRm, Carl Zeiss). Images from Hoechst 33,342 staining were quantified by scoring uniformly stained nuclei as healthy, while those with condensed or fragmented morphology were identified as pyknotic nuclei. The data were calculated as a percentage of cells with pyknotic nuclei and are presented as the mean ± SEM.

### Ex Vivo and In Vivo Study

#### Animals

All experimental procedures were approved by the Committee for Laboratory Animal Welfare and the Ethics Committee of the Maj Institute of Pharmacology PAS, Cracow, Poland.

All experiments were conducted on male Sprague–Dawley rats with an initial body weight of 225–250 g. The animals were kept in standard polyacrylic cages (5 animals/cage) with free access to water and standard laboratory food. Animals were kept at room temperature (22 °C) under an artificial light/dark cycle (12/12 h, light on at 7:00). The number of individuals was 5–6 per group.

#### Drugs and Treatments

1-Methyl-1,2,3,4-tetrahydroisoquinoline (1MeTIQ) was synthesized by the Department of Drug Chemistry, Maj Institute of Pharmacology PAS, Cracow, Poland. The purity of the compound was verified by the measurement of the melting point, and homogeneity was assessed on UPLC/MS methods. UPLC/MS analysis was performed on a Waters TQD spectrometer combined with UPLC Acquity H-Class with PDA eLambda detector. Waters Acquity UPLC BEH C18 1.7 μm 2.1 × 50 mm chromatographic column was used, at 40 °C, 0.3 mL/min flow rate, and 1.0 μL of injection volume (the samples were dissolved in LC–MS grade acetonitrile, typically at a concentration of 0.1–1 mg/mL prior to injection). All mass spectra were recorded under electrospray ionization in positive mode (ESI +) and chromatograms were recorded with UV detection in the range of 190–300 nm. The gradient conditions used were: 80% phase A (water + 0.1% formic acid) and 20% phase B (acetonitrile + 0.1% formic acid) to 100% phase B (acetonitrile + 0.1% formic acid) at 3.0 min, kept till 3.5 min, then to initial conditions until 4.0 min and kept for additional 2.0 min. Total time of analysis — 6.0 min. MK-801 (Sigma-Aldrich, USA) and 1MeTIQ were dissolved in a sterile 0.9% NaCl solution and injected in a volume of 1 mL/kg.

Saline, MK-801 (0.1 mg/kg), and 1 MeTIQ (25 or 50 mg/kg) were injected for 7 consecutive days between 10 a.m. and 11 a.m. In the combined groups (1MeTIQ + MK-801), 1MeTIQ was given 30 min before MK-801. On the last day of the experiment, drugs were given 2 h before decapitation.

The frontal cortex and hippocampus were dissected for further ex vivo analyses.

#### Microdialysis Study

Rats were anesthetized with ketamine (75 mg/kg) and xylazine (10 mg/kg) and secured in a stereotaxic frame (Stoelting, USA). Vertical microdialysis guide cannulas (Intracerebral Guide Cannula with stylet; BAS Bioanalytical, USA) were implanted in the hippocampus (HIP) according to the following stereotaxic coordinates: *A* / *P* − 5.5, L/M + 5.0 and *V* / *D* − 4.8 mm from bregma and the dura (G. Paxinos and C.H. Watson). Seven days after surgery, microdialysis probes (length 2 mm) were inserted into the cannulas, and the HIP was perfused with artificial cerebrospinal fluid (aCSF), which consisted of 140 mM NaCl, 2.7 mM KCl, 1.2 mM CaCl_2_, 1 mM MgCl_2_, 0.3 mM NaH_2_PO_4_, and 1.7 mM Na_2_HPO_4_ (Ph 7.4), at a flow rate of 1.5 μL/min maintained with a microinfusion pump (Stoelting, IL USA). Three basal samples were collected from freely moving rats at 30 min intervals after a 2 h wash-out period. Then, drugs (saline, MK-801 0.1 mg/kg, 1 MeTIQ 50 mg/kg) were injected. In the combined groups, 1MeTIQ was given 30 min before MK-801 injection. After drug administration, 6 experimental samples at 30 min intervals were collected. All dialysates were immediately frozen on dry ice (− 70 °C) until they were used in a biochemical assay.

#### Sample Preparation and High-Performance Liquid Chromatography Analysis

A 100 μL aliquot of mixed amino acid solution or sample, 175 μL of borate buffer solution, 200 μL of acetonitrile, and 25 μL of NBD-F working solution were mixed in a 1.5 mL centrifuge tube. The mixed solution was allowed to react at 60 °C in a water bath for 7 min, excluding light. NBD-F reacts with amino groups and enables amino acids to be detected with UV detection. After cooling to room temperature, 20 μL of the solution was injected into the equilibrated HPLC system.

The level of glutamate in dialysates (20 μL) was assayed using HPLC with a UV detector (472 nm), as described below. The Dionex Ultimate 3000 chromatograph (Coulochem III, Germany) was equipped with C18 columns (Hypersil Gold; 150 mm × 3 μm). The mobile phase comprised 0.02 M phosphate buffer (pH 6.0) with 16% acetonitrile. The flow rate was maintained at 0.75 mL/min. Chromatographic data were processed using the Chromeleon Dionex computer program (Germany). The levels of glutamate were quantified by calculating the height of the chromatograph peak and comparing it with a standard run on the day of the analysis.

#### Protein Measurement

The protein content in the rat brain homogenates (frontal cortex and hippocampus) was measured using the Pierce™ BCA Protein Assay Kit (catalog no. 23225, Thermo Fisher Scientific). All measurements were performed in duplicate.

#### Antioxidative Enzyme Activity Assay in the Frontal Cortex and Hippocampus

##### Glutathione Peroxidase

Brain tissue was homogenized in 7 mL of cold buffer (50 mM Tris–HCl, pH 7.5, 5 mM EDTA, and 1 mM DTT) per gram tissue and centrifuged (10,000 × g for 15 min at 4 °C). The supernatant was collected and analyzed using a Glutathione Peroxidase Assay Kit (cat. no. 703102, Cayman Chemical), following the manufacturer’s instructions. The results were calculated per mg of protein.

##### Glutathione Reductase

Brain tissue was homogenized in 7 mL of cold buffer (50 mM potassium phosphate, pH 7.5, 1 mM EDTA) per gram tissue and centrifuged (10,000 × g for 15 min at 4 °C). Supernatants were collected and analyzed using a Glutathione Reductase Assay Kit (cat. no. 703202, Cayman Chemical), following the manufacturer’s instructions. The results were calculated per mg of protein.

##### Catalase

Brain tissue was homogenized in 7 mL of cold buffer (50 mM potassium phosphate, pH 7.0 containing 1 mM EDTA) per gram tissue and centrifuged (10,000 × g for 15 min at 4 °C). Supernatants were collected and analyzed using a Catalase Assay Kit (cat. no. 707002, Cayman Chemical), following the manufacturer’s instructions. The results were calculated per mg of protein.

##### Superoxide Dismutase

Brain tissue was homogenized in 7 mL of cold 20 mM HEPES buffer, pH 7.2 (containing 1 mM EGTA, 210 mM mannitol and 70 mM sucrose) per gram tissue and centrifuged (1500 × g for 5 min at 4 °C). Supernatants were collected and analyzed using a Superoxide Dismutase Assay Kit (cat. no. 706002, Cayman Chemical) in accordance with the manufacturer’s instructions. The results were calculated per mg of protein.

#### Statistical Analysis

The results from the in vitro study after normalization were analyzed using Statistica software (StatSoft Inc., Tulsa, OK, USA). Analysis of variance (one- or two-way ANOVA) followed by Duncan’s post hoc test was used to show statistical significance at assumed *p* < 0.05. One-way ANOVA followed by post hoc Duncan’s test was used to compare ex vivo studies: antioxidative enzyme activity in the brain tissue. In vivo biochemical experiments (microdialysis study) were calculated by two-way repeated measures ANOVA, followed by Duncan’s post hoc test. All results were considered statistically significant when *p* < 0.05.

## Results

### In Vitro Study

**Fig. 2:**
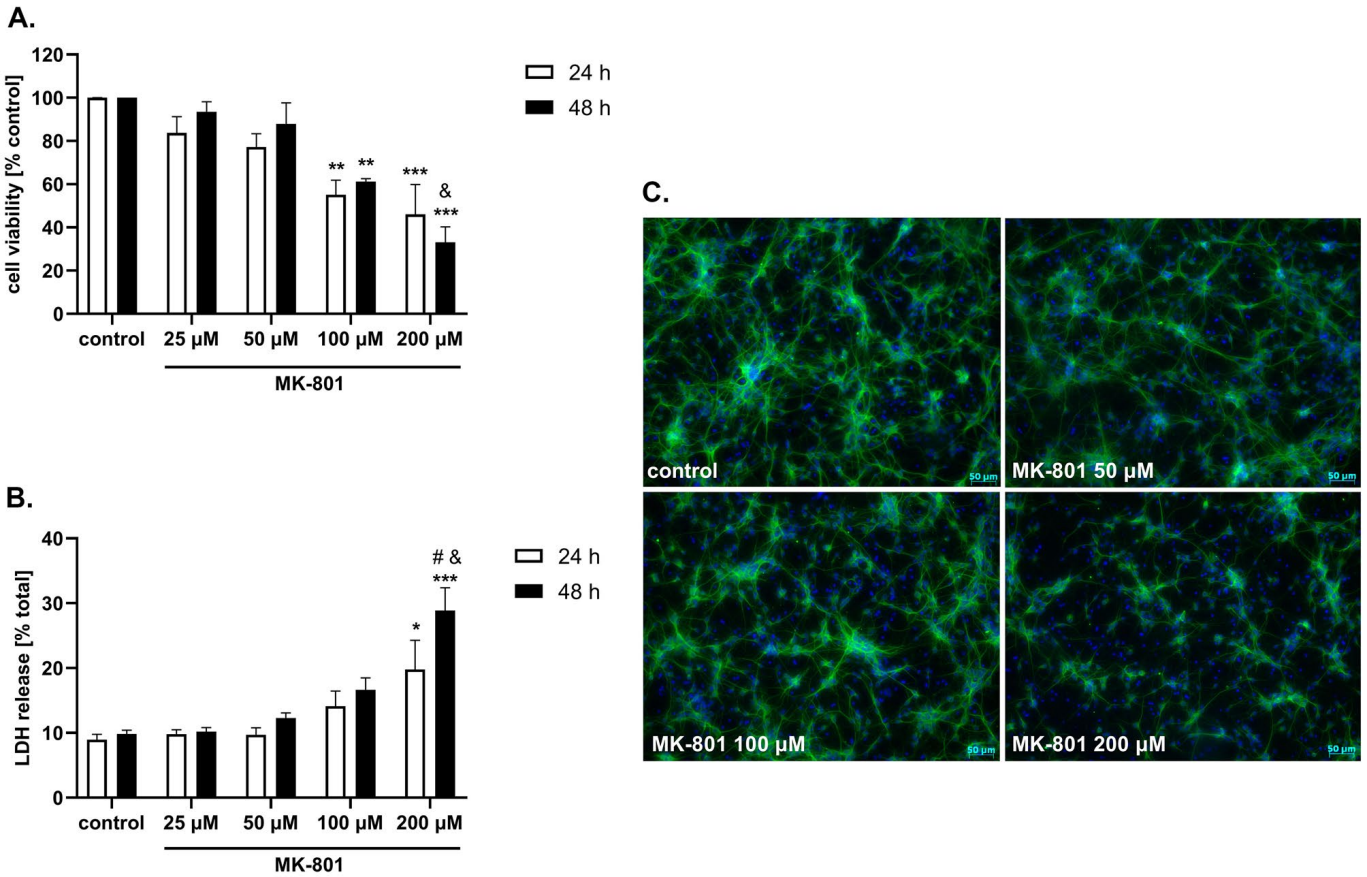
The effect of MK-801 in 3 DIV (*days *in vitro) primary neuronal cell cultures. The cells were treated with MK-801 (20, 50, 100 and 200 μM) for 24 and 48 h. (Panel **A**). Results of cell viability measured by MTT reduction assay. Data were normalized to vehicle-treated cells (control) and are presented as the mean ± SEM from 2 independent experiments. (Panel **B**). Results of cell toxicity measured by LDH release assay. Data were normalized to Triton X100-treated cells (total damage) and are presented as the mean ± SEM from 2 independent experiments. Data were analyzed by two-way ANOVA with Tukey’s post hoc test. ** *p* < 0.01 and *** *p* < 0.001 vs. vehicle-treated cells; ^&^*p* < 0.05 and ^&&^*p* < 0.01 200 μM vs. 100 μM; ^#^*p* < 0.05 48 h vs. 24 h. (Panel **C**) Representative microphotographs of neuronal cells treated for 24 h with MK-801 (50, 100, and 200 μM). After treatment, the cells were fixed and stained with neuronal (antiMAP2, green) and nuclear (Hoechst 3342) markers

MK-801 at concentrations of 100 and 200 μM evoked a significant reduction in neuronal cell viability after 24 and 48 h of treatment (approx. 40% and 60%, respectively). Two-way ANOVA revealed a concentration-dependent effect of MK-801 at concentrations of 100 and 200 μM after 48 h of treatment; however, no significant time dependency was found (Fig. 2A). The cell damaging effect of 200 μM MK-801 was accompanied by induction of cytotoxicity, as shown by LDH release assay, where this agent evoked a significant increase in LDH release after 24 and 48 h of treatment (approx. 2- and threefold compared to the basal level, respectively) (Fig. 2B). Two-way ANOVA revealed a time- and concentration-dependent effect of MK-801 in the LDH release assay (Fig. 2B). The detrimental effect of MK-801 on neurons was also confirmed morphologically, where this compound at concentrations of 100 and 200 μM significantly decreased the number of neurons (antiMAP-2 immunocytochemistry) (Fig. 2C) and increased the number of damaged nuclei (Hoechst 33,342 staining) (Fig. 2C).

**Fig. 3:**
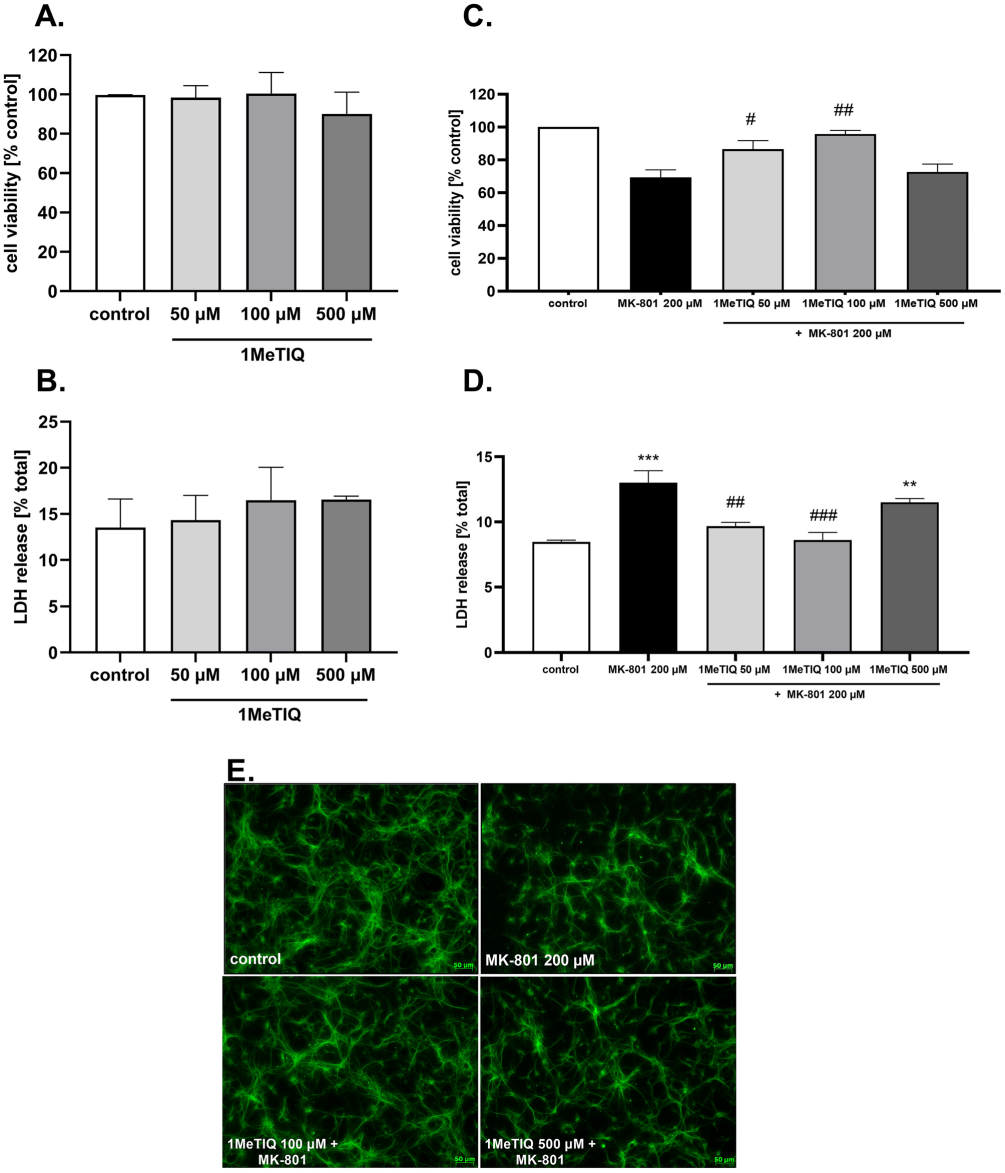
The effect of 1MeTIQ alone and on MK-801-induced cell damage in primary neuronal cell cultures. The cells were treated for 24 h with 1MeTIQ alone (50, 100, and 500 μM) or with 1MeTIQ (10, 50, 100, and 500 μM) in combination with MK-801 (200 μM) for 24 h. (Panels **A** and **C**) Results of cell viability measured by MTT reduction assay. Data were normalized to vehicle-treated cells (control) and are presented as the mean ± SEM from 3 independent experiments. (Panels **B** and **D**). Results of cell toxicity measured by LDH release assay. Data were normalized to Triton X100-treated cells (total damage) and are presented as the mean ± SEM from 3 independent experiments. Data were analyzed by one-way ANOVA with Tukey’s post hoc test. ** *p* < 0.01 and *** *p* < 0.001 vs. vehicle-treated cells; ^#^*p* < 0.05, ^##^*p* < 0.01 and ^###^*p* < 0.001 vs. MK-801-treated cells. (Panel **E**). Representative microphotographs of neuronal cells treated for 24 h with 1 MeTIQ (100 and 500 μM) and MK-801 (200 μM). After treatment, the cells were fixed and stained with neuronal (antiMAP2, green) markers

**Fig. 4:**
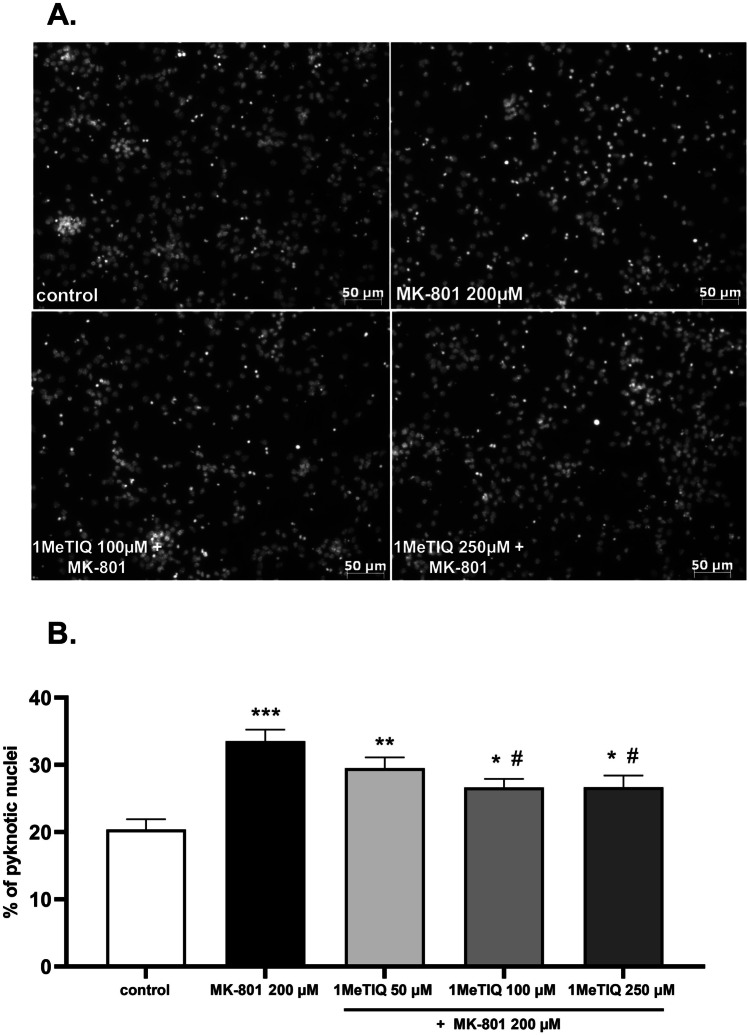
The effect of 1MeTIQ on MK-801-induced nuclei damage. (Panel **A**) Representative microphotographs of primary neuronal cells treated for 24 h with 1MeTIQ (100 and 250 μM) and MK-801 (200 μM) stained with Hoechst 33,342. (Panel **B**) Histograms showing the number of pyknotic nuclei after 24 h of cell treatment with 1 MeTIQ (50, 100, and 250 μM) and MK-801 (200 μM). Data were calculated as the number of damaged nuclei to total ones and are presented as the mean ± SEM from 2 independent experiments. Data were analyzed by one-way ANOVA with Tukey’s post hoc test. * *p* < 0.05, ** *p* < 0.01 and *** *p* < 0.001 vs. vehicle-treated cells; ^#^*p* < 0.05 vs. MK-801-treated cells

1MeTIQ given alone at all concentrations (50, 100, and 500 μM) was safe for neuronal cells, as confirmed by MTT reduction (Fig. 3A) and LDH release (Fig. 3B) assays after 24 h of treatment. 1MeTIQ at concentrations of 50 and 100 μM but not 500 μM significantly prevented the MK-801 (200 μM)-induced reduction in cell viability (Fig. 3C) and LDH release (Fig. 3D). The neuroprotective effect of 1MeTIQ was also confirmed at the morphological level (antiMAP2 immunochemistry), where this compound at a concentration of 100 μM but not 500 μM increased the number of surviving neurons after 24 h of treatment with MK-801 (Fig. 3E). Moreover, we showed a significant increase in the number of pyknotic (fragmented or condensed) nuclei after MK-801 (200 μM) treatment (approximately 15% when compared to the vehicle-treated group), which was partially attenuated by 1MeTIQ at concentrations of 100 and 250 μM (Fig. 4A, B).

### Ex Vivo Study — Frontal Cortex

#### Glutathione Peroxidase

**Fig. 5:**
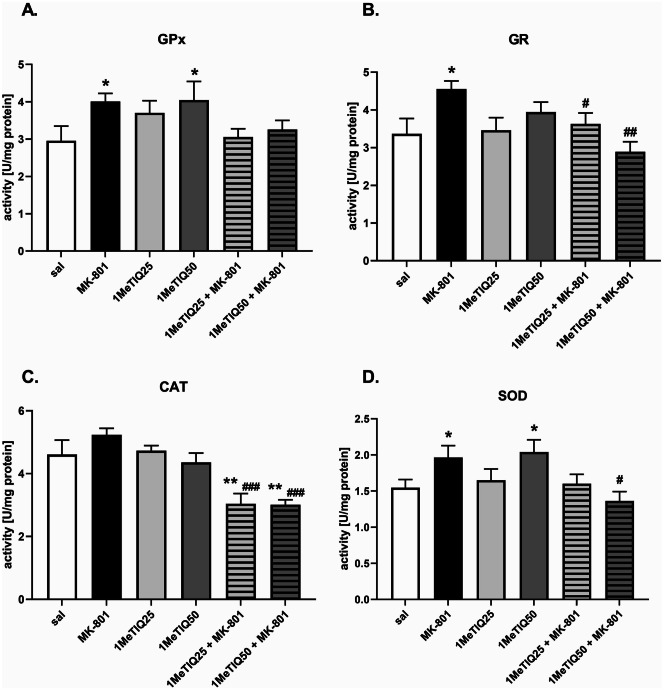
The effect of 1MeTIQ and MK-801 on the activity of antioxidant enzymes (GPx, GR, CAT, and SOD) in the rat frontal cortex (Fcx). The results are expressed as the means ± SEM from 5–6 different samples using homogenates from the different parts of 5–6 rat brains. Data were analyzed by one-way ANOVA with Duncan’s post hoc test. * *p* < 0.05, ** *p* < 0.01 vs. saline/control group; ^#^*p* < 0.05, ^##^*p* < 0.01 vs. MK-801 group

One-way ANOVA showed a significant effect (*F* [5,26] = 3.09, *p* < 0.05) of the treatment on glutathione peroxidase activity. Post hoc tests showed a significant increase in GPx activity after MK-801 or 1 MeTIQ (50 mg) was given alone (both *p* < 0.05) compared to saline (Fig. 5A).

#### Glutathione Reductase

One-way ANOVA revealed a significant effect (*F* [5,26] = 3.67, *p* < 0.05) of the applied treatment on GR activity. Duncan’s post hoc test showed a significant (*p* < 0.05) increase in GR activity after MK-801 treatment compared to saline. In the combined groups, both doses of 1MeTIQ (25 and 50 mg) reversed the effect of MK-801 and decreased GR activity (*p* < 0.05 and *p* < 0.01, respectively) (Fig. 5B).

#### Catalase

Statistical analysis showed a significant effect (*F* [5,24] = 9.26, *p* < 0.001) of the treatment on CAT activity. Post hoc analysis revealed changed CAT activity after combined treatments: 1MeTIQ (25 or 50 mg) given together with MK-801 significantly decreased enzyme activity compared to saline (both *p* < 0.01) or MK-801-treated animals (both *p* < 0.001) (Fig. 5C).

#### Superoxide Dismutase

One-way ANOVA showed a significant effect (*F* [5,28] = 3.15, *p* < 0.05) of the treatment on SOD activity. Both MK-801 and 1MeTIQ (50 mg) given alone significantly (*p* < 0.05) increased SOD activity compared to saline. 1MeTIQ (50 mg) administered together with MK-801 significantly (*p* < 0.05) decreased enzyme activity compared to that of the MK-801-treated group (Fig. 5D).

### Ex Vivo Study — Hippocampus

#### Glutathione Peroxidase

**Fig. 6:**
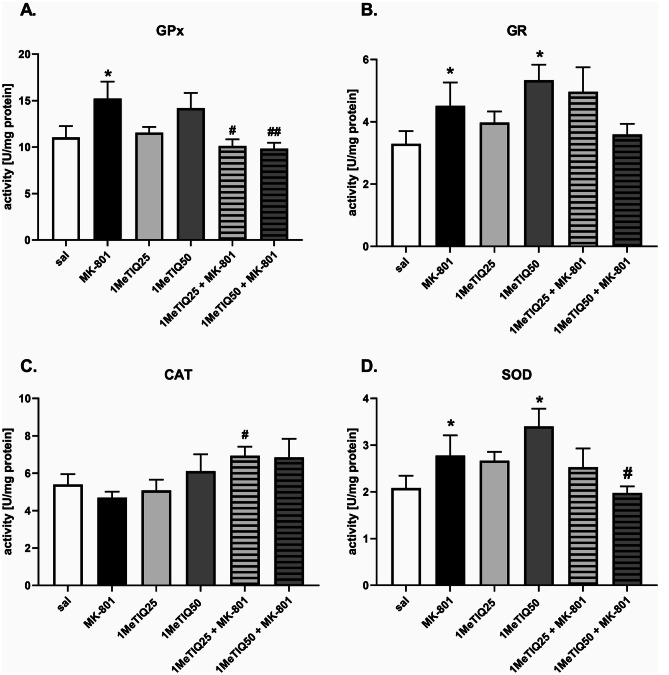
The effect of 1MeTIQ and MK-801 on the activity of antioxidant enzymes (GPx, GR, CAT, and SOD) in the rat hippocampus (HIP). The results are expressed as the means ± SEM from 5–6 different samples using homogenates from the different parts of 5–6 rat brains. Data were analyzed by one-way ANOVA with Duncan’s post hoc test. * *p* < 0.05 vs. saline/control group; ^#^*p* < 0.05, ^##^*p* < 0.01 vs. MK-801 group

One-way ANOVA showed a significant effect (*F* [5,22] = 3.28, *p* < 0.05) of the applied treatment on GPx activity in the hippocampus. Post hoc Duncan’s test showed significantly (*p* < 0.05) increased GPx activity after MK-801 treatment compared to saline. In the combined groups, 1MeTIQ (25 or 50 mg) administered together with MK-801 reversed this effect of MK-801 and significantly (both *p* < 0.05) decreased GPx activity (Fig. 6A).

#### Glutathione Reductase

One-way ANOVA showed a significant effect (*F* [5,25] = 3.13, *p* < 0.05) of the treatment on GR activity. Post hoc analysis revealed a significant (*p* < 0.05) increase in GR activity after MK-801 or 1 MeTIQ (50 mg) treatment compared to saline (Fig. 6B).

#### Catalase

One-way ANOVA revealed no significant effect (*F* [5,22] = 2.04, *p* = *n. s.)* of the applied treatment on CAT activity in the hippocampus (Fig. 6C).

#### Superoxide Dismutase

One-way ANOVA showed a significant effect (*F* [5,28] = 2.67, *p* < 0.05) of the treatment on SOD activity. Post hoc analysis revealed a significant (*p* < 0.05) increase in SOD activity after MK-801 or 1 MeTIQ (50 mg) was given alone compared to the control group. In the combined groups, 1MeTIQ (50 mg) given together with MK-801 significantly (*p* < 0.05) decreased SOD activity, restoring it to the control level (Fig. 6D).

#### In Vivo Microdialysis Study

**Fig. 7:**
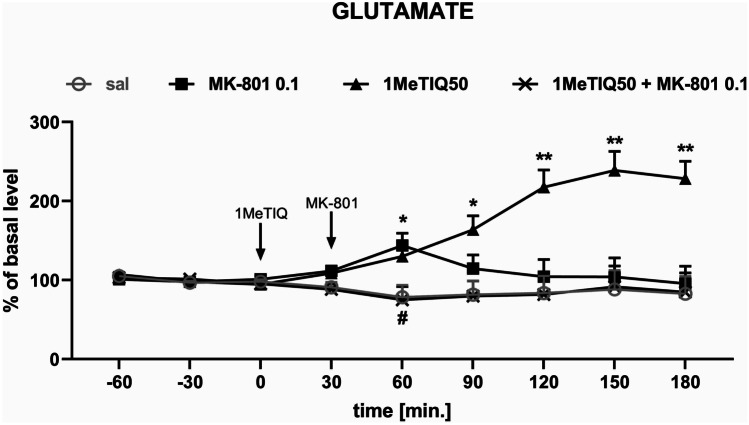
The effect of 1MeTIQ (50 mg/kg i.p.) and MK-801 (0.1 mg/kg i.p.) on glutamate release in the rat hippocampus — an in vivo microdialysis study. 1MeTIQ was injected at time point “0”, while MK-801 was injected 30 min later. The values are the mean ± SEM. The number of animals (*n* = 5–6 rats in a group) is expressed as a percent of the basal level. An average concentration of three stable samples prior to drug administration was regarded as a control value and was considered to be 100%. Data were analyzed by two-way ANOVA for repeated measures followed by Duncan’s post hoc test. * *p* < 0.05, ** *p* < 0.01 vs. saline/control group; ^#^*p* < 0.05 vs. MK-801 group

The mean control basal extracellular concentration of glutamate in dialysates obtained from the hippocampus was approximately 1.1 ± 0.3 (pg/20 μL). Two-way repeated-measures ANOVA demonstrated a significant effect of treatment (*F*[3,19] = 8.01; *p* < 0.01) on glutamate release in the rat hippocampus. Post hoc test showed an increase (approx. 150%) in glutamate concentration in the synaptic cleft 60 min after acute MK-801 (0.1 mg/kg i.p.) administration (Fig. 7). The same analysis indicated that acute 1MeTIQ administration produced a significant elevation in glutamate release (up to 250%; 90–180 min.). In the combined treatment with MK-801 and 1MeTIQ, the glutamate concentration returned below the control (saline) value (Fig. 7).

## Discussion

The results obtained in the present study indicated that MK-801 exhibits dose-dependent neurotoxic properties. In vitro studies clearly show that MK-801 at the highest concentrations has strong cytotoxic potential, while ex vivo studies demonstrated that MK-801 administered to rats at a low dose (0.1 mg/kg i.p.) induces mild oxidative stress and a small increase in glutamate release in the rat hippocampus. On the other hand, 1MeTIQ administered at a higher dose (50 mg/kg i.p.) shows a limited neuroprotective effect and partially reverses the toxic effects induced by MK-801.

There is evidence that the effect of MK-801 depends on the dose used and the route of administration. When used at low concentrations, it has neuroprotective properties (Radesäter et al. 2003). For example, MK-801 attenuated neuronal damage caused by 6-OHDA (Massari et al. 2016), oxygen–glucose deprivation (Domin et al. 2015), or glutamate (Jantas et al. 2018). A high dose of this NMDA receptor antagonist is highly toxic and leads to the death of neurons by apoptosis (Zhang et al. 1996; Alavez et al. 2006) or necrosis (Willis and Ray 2007). Moreover, it has been shown that in various in vitro models MK-801 could evoke neuronal cell death by apoptotic and necrotic mechanisms (Peng et al. 2013; Zhu et al. 2016). The results obtained by us in in vitro experiments fully confirm these data. MK-801 shows toxic activity in cultures of forebrain neurons at the highest concentrations, i.e., 100 and 200 µM (Fig. 2A, B). At these high concentrations, MK-801 causes a strong decrease in the survival of nerve cells and at the same time causes a strong increase in the release of LDH. Moreover, using the Hoechst 33,342 staining method, MK-801 at concentrations of 100 and 200 µM caused an increased number of damaged nuclei (Fig. 2C). As shown in our study, concentration- and time-dependent effects of MK-801 in LDH release assays suggest the involvement of apoptosis and probably secondary necrosis. This was also confirmed by the measurement of the number of pyknotic nuclei where fragmented (apoptotic) and condensed (necrotic) nuclei are visible (Fig. 4A). Finally, the MK-801-induced increase in the number of pyknotic nuclei was attenuated by caspase-3 inhibitor, Ac-DEVD-CHO (data not shown) pointing to apoptosis as a main executor of MK-801-mediated neuronal cell damage.

1MeTIQ, which is a natural antioxidant, given alone does not affect cell viability or the amount of LDH released (Fig. 3A, B). However, two concentrations of 1MeTIQ (50 and 100 µM) administered together with MK-801 (200 µM) showed a neuroprotective action, completely inhibiting its toxic effects and returning both tested parameters to the control level (Fig. 3C, D). The highest concentration of 1MeTIQ (500 µM) did not have a neuroprotective effect on the toxicity induced by MK-801 (Fig. 3C, D). Similar effects were observed using Hoechst 33,342 staining, where 1MeTIQ at a concentration of 100 µM given together with MK-801 had a protective effect. However, at a high concentration, 500 µM 1MeTIQ did not show such an effect (Fig. 3E). Moreover, we indicated a significant increase in the number of damaged nuclei after MK-801 (200 μM) treatment, which was partially attenuated by 1MeTIQ at concentrations of 100 and 250 μM (Fig. 4A, B). LDH was originally recognized as a necrosis marker (Koh and Choi 1987), and in the 1990s, it was found to accurately measure neuronal apoptosis in cell culture since in the absence of microglia cells, neurons die by secondary necrosis (Koh and Cotman 1992). The loss of intracellular LDH and its release into the culture medium are indicators of irreversible cell death due to cell membrane damage. The LDH leakage assay is based on the release of the enzyme into the culture medium after cell membrane damage, whereas the MTT assay is mainly based on the enzymatic conversion of MTT in the mitochondria and has been used to accurately measure neuronal injury (Loo and Rillema 1998). All the present results obtained by us in in vitro studies clearly show that 1MeTIQ has neuroprotective potential and protects nerve cells from the toxic effects induced by MK-801. The above results confirm our previous studies in which we showed that 1MeTIQ blocked the production of free radicals and the neurotoxicity induced by glutamate (it inhibited both caspase 3 activity and LDH release and prevented glutamate-induced cell death and excitotoxicity caused by ^45^Ca^2+^ influx) (Antkiewicz-Michaluk et al. 2006). Other authors indicated that 1MeTIQ decreased ROS levels and prevented the loss of synaptic proteins (e.g., NR1 NMDAR subunit, PSD-95, and synaptophysin expression) induced by amyloid β in primary neuronal culture model systems. In addition, 1MeTIQ reduced H_2_O_2_-induced ROS production in immature hippocampal neuronal cultures. All in vitro results support the hypothesis that 1MeTIQ is an NMDAR antagonist with strong antiradical activity (Kuszczyk et al. 2014). Moreover, it was demonstrated that 1MeTIQ inhibited the binding of radioactive [^3^H]MK-801 to isolated brain membranes (Antkiewicz-Michaluk et al. 2006). Most likely, due to this mechanism (blocking MK-801 binding to the cell membrane), 1MeTIQ reduces/weakens the toxic effect of MK-801.

It is well known that oxidative stress is one of the factors implicated in the pathogenesis of schizophrenia (Mahadik and Mukherjee 1996). Because ROS are involved in membrane pathology, they may cause neuronal injury and, consequently, cell death (Akyol et al. 2002; Mahadik et al. 2006). It is well known that oxidative stress is a universal pathogenic mechanism leading to cell death, which may result from excitotoxicity, mitochondrial dysfunction, and/or excessive dopamine catabolism by MAO (Obata 2002; Forder and Tymianski 2009; Federico et al. 2012). There is evidence that treatment with MK-801 generates ROS (Willis and Ray 2007). Our ex vivo study demonstrated that a low dose of MK-801 significantly increased the activity of three antioxidant enzymes (GPx, GR, and SOD) in both the frontal cortex and hippocampus (Figs. 5 and 6). Only the CAT activity was unchanged in this group. The increase in the activity of antioxidant enzymes, both in the frontal cortex and in the hippocampus, indicates that oxidative stress takes place in neurons and the cellular defense reaction.

Similar results were observed by other authors using a higher dose of MK-801: after treatment with a high dose of MK-801 (0.5 mg/kg i.p.) the level of malondialdehyde (MDA), an indicator of lipid peroxidation, and the activity of antioxidant enzymes such as SOD and GPx were increased significantly in the prefrontal cortex compared to the control group, whereas CAT activity was not changed. Moreover, in the MK-801 groups, a great number of apoptotic cells were observed (Ozyurt et al. 2007). Some authors have indicated that CAT is not the primary factor controlling oxidative stress (Vernet et al. 2004), and its role is important in the overexpression of SOD (Przedborski et al. 1992) when the concentration of H_2_O_2_ is higher than 10^–6^ M (Cohen and Hochstein 1963). SOD catalytic activity leads to the conversion of superoxide radicals to H_2_O_2_, while the catalytic activity of GPx transforms it in water. GPx is the only enzyme capable of eliminating H_2_O_2_ from mitochondria. On the other hand, GR acts as a GPx cofactor and has antioxidant and free radical scavenger properties (Imai and Nakagawa 2003).

Our current results show that 1MeTIQ administered in combination with MK-801 restores the activity of antioxidant enzymes to the control level in both measured brain structures (Figs. 5 and 6). Earlier data indicate that 1MeTIQ is a natural antioxidant with a very interesting mechanism of action. First, 1MeTIQ is an inhibitor of the MAO enzyme, which strongly inhibits the process of dopamine oxidation and, at the same time, minimizes the production of ROS in dopamine neurons (Antkiewicz-Michaluk et al. 2003, 2004). Second, there is evidence that 1MeTIQ protects the activity of NADH–ubiquinone oxidoreductase enzyme, suppresses the inhibition by MPP + of mitochondrial respiratory complex I and thus prevents the ROS-mediated neurotoxic effect of MPP + (Parrado et al. 2000). Third, 1MeTIQ is an NMDAR antagonist and may indirectly inhibit excitotoxicity-evoked ROS production (Kuszczyk et al. 2014).

NMDA receptors are located throughout the brain, but the highest levels of these receptors are found in the CA1 region of the hippocampus (Watson and Stanton 2009; Jafari-Sabet 2011). It is well documented that the hippocampus is involved in the memory and learning of humans and animals (e.g., declarative memory, emotions, and spatial learning) (Rezayof et al. 2010; Lu et al. 2011; Khakpai et al. 2012, 2013). The NMDA receptor complex is an ionic channel and plays a key role in regulating the length of excitatory postsynaptic potential and, consequently, in synaptic plasticity and cognitive functions and triggering excitotoxic processes leading to the death of neurons (Alavez et al. 2006; Khakpai et al. 2016). One of the main effects of blocking NMDA receptors is the excessive release of glutamate (Moghaddam et al. 1997; Adams and Moghaddam 1998) in multiple brain regions. It has been proposed that overstimulation of postsynaptic neurons might cause cognitive and behavioral disturbances associated with the NMDA receptor hypofunction state (Olney and Farber 1995; Moghaddam et al. 1997; Adams and Moghaddam 1998). Another important consequence of NMDA receptor blockade is the decrease in recurrent local feedback inhibition (Grunze et al. 1996). There is evidence that NMDA antagonists may preferentially change NMDA-dependent modulation of recurrent local circuit inhibition in the hippocampus (Grunze et al. 1996).

The present results from an in vivo microdialysis study showed that acute treatment with MK-801 at a low dose (0.1 mg/kg i.p.) produced weak and short-term increases in glutamate release in the rat hippocampus (Fig. 7). In the same experiment, 1MeTIQ given alone at a dose of 50 mg/kg also induced elevation of glutamate release, while given combined with MK-801 completely inhibited the increase in extraneuronal hippocampal glutamate levels induced by MK-801 administration (Fig. 7). A similar effect was observed in our previous studies performed in the rat frontal cortex, where 1MeTIQ completely antagonized the increase in glutamate release after MK-801 administration (Pietraszek et al. 2009). Moreover, a previous in vivo microdialysis experiment demonstrated that 1MeTIQ also prevents kainate-induced release of glutamate and aspartate from the rat frontal cortex (Antkiewicz-Michaluk et al. 2006).

The results from microdialysis studies revealed that complex neural circuitry may be involved in neurotoxicity induced by NMDA receptor hypofunction (Kim et al. 1999; Farber et al. 2002). Glutamate plays a key role in this circuit as a regulator of inhibitory tone by tonically stimulating NMDA receptors on GABAergic interneurons, which, in turn, inhibit excitatory projections to cerebrocortical neurons. NMDA receptor antagonists prevent glutamate from driving GABAergic inhibitory neurons, resulting in a loss of inhibitory control over excitatory projections to the cerebral cortex (Newcomer et al. 2000).

In conclusion, in the present paper, we demonstrated that an uncompetitive antagonist of the NMDA receptor, MK-801, at high concentrations shows toxic activity in primary neuronal cell cultures which was attenuated by 1MeTIQ. Moreover, MK-801 administered at a low dose to rats causes an increase in oxidative stress in both the frontal cortex and the hippocampus. In addition, in in vivo microdialysis studies, a low dose of MK-801 caused an increase in glutamate release in the rat hippocampus. 1MeTIQ administered at a higher dose (50 mg/kg i.p.) shows a limited neuroprotective effect and partially reverses the toxic effects induced by MK-801.

## Abbreviations

CAT: Catalase
COMT: Catechol-O-methyltransferase
Fcx: Frontal cortex
GABA: Gamma-aminobutyric acid
GPx: Glutathione peroxidase
GR: Glutathione reductase
HIP: Hippocampus
HPLC: High-performance liquid chromatography
LDH: Dehydrogenase leakage
MAO: Monoamine oxidase
1MeTIQ: 1-Methyl-1,2,3,4-Tetrahydroisoquinoline
MK-801: Dizocilpine
MPP^+^: 1-Methyl-4-phenylpyridinium ion
MTT: M tetrazolium
NMDA: N-methyl-D-aspartate
PD: Parkinson’s disease
PPI: Prepulse inhibition
ROS: Reactive oxygen species
SOD: Superoxide dismutase
